# Controlled Dry Adhesion of Bio-Inspired Fibrillar Polymers: Mechanics, Strategies, and Recent Advances

**DOI:** 10.3390/ma18071620

**Published:** 2025-04-02

**Authors:** Shuo Xu, Anahita Emami, Meysam Khaleghian

**Affiliations:** 1 Ingram School of Engineering, Texas State University, San Marcos, TX 78666, USA; uzr14@txstate.edu; 2 Department of Engineering Technology, Texas State University, San Marcos, TX 78666, USA

**Keywords:** bio-inspired Microstructures, frictional adhesion, microscale contact mechanics, nonlinear adhesion mechanics

## Abstract

Recent advancements in tunable adhesion technologies have broadened the scope of applications for bio-inspired fibrillar adhesives. This review highlights the latest developments in controlled adhesion mechanisms, with a focus on bio-inspired fibrillar systems. We examine key theoretical foundations and progress in controllable adhesion, including contact mechanics, contact splitting efficiency, fracture mechanics, and the interplay between adhesion and friction. Various factors influencing adhesion strength are discussed alongside optimization approaches and innovative designs that enhance performance. The review also covers recent research on switchable adhesion strategies, with an emphasis on methods for regulating surface contact, stress distribution, and shear force control. Finally, we identify the primary challenges and future directions in the field, outlining areas that require further exploration and technological development. This paper aims to provide a comprehensive overview of current advancements and offer insights to guide future research in the evolving field of tunable adhesion technologies.

## 1. Introduction

Geckos can climb flexibly in a variety of environments. This exceptional ability is due to the unique structure of the gecko’s feet, which allows for rapid conversion between attachment and detachment through the micro-nano structure of the toe surface. Inspired by this, many attempts have been made to create synthetic fibrillar adhesives, especially reversible adhesives, for developing artificial attachment devices. These attempts range from designing asymmetric or mushroom-shaped microstructures to using activatable adhesion. Asymmetric microstructures enable anisotropic behavior in normal and shear attachment, while mushroom-shaped microstructures offer stronger adhesion. Actuation for switching from an adhesive to a non-adhesive state is achieved through an external force field or through embedded active materials that respond to an external stimulus. For practical applications in artificial attachment devices, strong attachment and rapid, easy switching between attachment and detachment are crucial for achieving controlled adhesion.

Since 2000, gecko adhesion has been studied intensively. Many of the basic principles allowing for the enhancement and control of adhesion are well understood today. For example, at the fibril level, the mechanics of fibrillar adhesives are influenced by the size and shape of the fiber, as well as the material properties such as elastic modulus, material gradients, and composite fibrils. However, the elastic coupling between the fibrils plays an important role in the effective control of the adhesion and detachment of artificial attachment systems. After a comprehensive research effort, contact splitting, shape modification, and gradient strategies at the fibril level are generally accepted as efficient means of enhancing adhesion and making it controllable. In the last decade, research has focused on the elastic coupling and interactions among fibrils, mediated through the array’s backing layer, load distribution among the fibrils, and the statistics of individual fibril detachment [1]. These advances have paved the way for the efficient exploitation of adhesive effects in emerging applications, such as robotic gripper [2,3], in-space grasping and manipulation [4,5,6,7], transfer printing [8], wall-climbing robots [9,10], and human climbing [11].

Over the past two decades, the literature on adhesion mechanisms and novel attachment devices has grown substantially. Recently, several excellent reviews have thoroughly investigated switching mechanisms, triggers, releasing strategies, measurement techniques, and applications. It is worth citing these reviews as foundational work for the present study. In a feature article, Hensel et al. [12] reviewed the roles of contact geometry and mechanical properties in controlling the adhesive performance of synthetic micropatterned adhesives. Another review by Croll et al. [13] focused on the underlying switching mechanisms, triggers, and measurement techniques. Arzt et al. [1] provided a broader review of functional surface microstructures inspired by nature, discussing the latest developments in adhesion modulation by functional surface microstructures up to 2021. Meiners and Tracht [14] listed the releasing strategies for adhesive grasping systems and evaluated these strategies with respect to the specific requirements, concluding that a switchable microstructure and a peeling strategy are the most suitable releasing approaches. More recently, Duan et al. [15] reviewed the adhesion mechanisms of three types of biological organisms (gecko, tree frog, and octopus) alongside their corresponding artificial adhesive surfaces, examining soft contact interfaces and interfacial forces between micro/nanostructures and substrates. Similarly, Liu et al. [16] reviewed structures, fabrication methods, and applications of gecko-inspired controllable adhesives. Zhao et al. [17] provided a comprehensive review of the progressive evolution in adhesive research, transitioning from strength-focused to smart-actuated adhesives. These reviews provide valuable context for the current work.

Developments in this field are rapid and extensive, making it difficult to cover all aspects in a single article. This paper discusses tunable adhesion technologies in bio-inspired fibrillar adhesives, focusing on recent advancements—primarily from the past five years—in interface mechanics from individual fibrils to micropatterned systems, optimization approaches to enhance adhesion, new designs to improve performance, and strategies for achieving switchable adhesion. Accordingly, in Section 2, we summarize relevant theories and fundamentals of controllable adhesion and research progress, including advances in contact splitting, fracture mechanics at the interface between microfibrils and substrates, and coupled friction and adhesion. Section 3 reviews recent research on factors affecting effective adhesion strength. In Section 4, we examine innovative designs and optimizations of fibrillar adhesives, as well as recent progress and case studies on controlling adhesion strategies from a mechanics perspective. In Section 5, we discuss key challenges in bio-inspired adhesives, including interface mechanics, environmental reliability and durability, repeatability and precision control, and the expansion of applications into medical devices. Finally, in Section 6, we briefly outline possible research areas or problems to be addressed from mechanics and reversible adhesion control perspectives.

## 2. Interface Mechanics Between Microfibrils and Substrate

**Figure 1:**
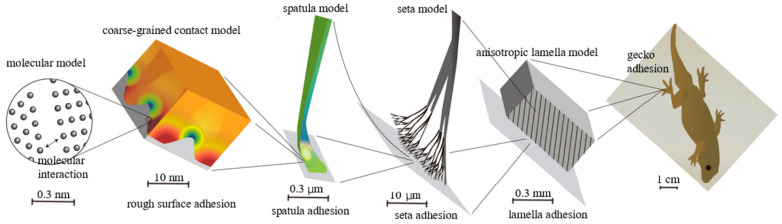
Multiscale modeling hierarchy of the adhesion mechanism used by the gecko (redrawn based on [18]).

From a mechanics perspective, the material and structural properties of gecko foot pads play an essential role in their adhesion capabilities across multiple scales. Sauer [18] reviewed computational models of the gecko adhesion mechanism, as illustrated in Figure 1. Studies by Autumn et al. [19] and Russell et al. [20] have revealed how geckos effectively deploy this complex, multiscale system. Several fundamental mechanical principles have emerged from these investigations. In this section, we will briefly discuss the basic mechanics of attachment and detachment in fibrillar adhesive systems.

### 2.1. Contact Mechanics and Contact Splitting Efficiency

#### 2.1.1. Pull-Off Force and Peel Force

The gecko’s adhesion system operates based on physical principles largely independent of the chemistry of the two interacting surfaces. Van der Waals forces are the primary mechanism of adhesion in gecko setae [21]. Since van der Waals forces are minimally affected by temperature, pressure, humidity, or external electromagnetic fields, gecko-inspired dry adhesives are suitable for a wide range of environmental conditions. For elastic contact under adhesion, the pull-off force between a sphere of radius *R* and a plane can be described by the JKR (Johnson–Kendall–Roberts) model of contact mechanics [22]:

(1) $$F_{pull-off}=\frac{3}{2}\pi R\Delta \gamma$$

where Δγ is the adhesion energy per unit area given by

(2) $$\Delta \gamma =\gamma_{1}+\gamma_{2}-\gamma_{12}$$

where *γ*_1_ and *γ*_2_ are the surface energies of the two materials, respectively, and *γ*_12_ is the interfacial energy. According to this model, for a given thermodynamic adhesion energy Δγ, a smaller radius *R* results in a greater adhesive force per unit area. The JKR model offers insights into enhancing contact area and adhesion via elastic deformation, which is valuable for designing the adhesive strength of synthetic gecko-inspired systems. However, this model does not fully account for the complex hierarchical structures of gecko setae or the sophisticated peeling and detachment mechanisms that enable rapid and efficient foot detachment. The peeling model more directly explains controlled detachment, focusing on energy release rates and the influence of peel angle.

For a single spatula inclined relative to the surface normal, the peel force, *F_peel_*, can be analyzed using Kendall’s tape peeling model [23] as follows:

(3) $$G=\frac{F_{peel}}{b}(1-\cos\theta )+{\left(\frac{F_{peel}}{b}\right)}^{2}\frac{1}{2E^{*}h}=\Delta \gamma$$

where *b* is the width of the tape, *θ* is the peel angle, *h* is the thickness of the tape, and $E^{*}=E/\left(1-\nu^{2}\right)$ is the plane strain modulus of the tape. *G* is the energy release rate of a growing crack under steady-state conditions. The Kendall model describes how the peel angle, the tape configuration, and stiffness influence peeling strength. For an uneven substrate, the peel force can be expressed as [24].

(4) $$\frac{F_{peel}}{b}=hE\left\{\cos\theta \cos\alpha -1+\frac{1}{2}\left[\sqrt{{\left(\cos(\theta +\alpha )-1\right)}^{2}+\frac{2G}{hE}}+\sqrt{{\left(\cos(\theta -\alpha )-1\right)}^{2}+\frac{2G}{hE}}\right]\right\}$$

where α is a small perturbation angle defined by the surface topography. The adhesive energy Δγ in Kendall’s peel theory can be extended to incorporate the effects of shear and normal tractions [25,26,27,28]. For a comprehensive review of common models describing peeling in biological and bio-inspired adhesive systems, see [29]. The Kendall peeling model provides insights into achieving efficient, controlled detachment—crucial for designing high-performance gecko-inspired adhesive systems. By integrating JKR principles with peeling mechanics or hierarchical structures that mimic the controlled peeling seen in gecko feet, these systems can achieve strong adhesion with easy detachment.

#### 2.1.2. Contact Splitting Efficiency

If a contact area, *A_total_*, is divided into *n* sub-contacts, the pull-off force or peel force after contact splitting is given by [30]

(5) $$F^{\prime }=n^{s}F$$

where *s* is the contact splitting efficiency, varying with different contact geometries.

(6) $$s=\frac{\sum_{i=1}^{n}A_{i}}{A_{total}}$$

where *A_i_* is the effective contact area of a single fiber. Higher splitting efficiency indicates more efficient load distribution and contact adaptability. This efficiency varies with different contact geometries. For instance, the corresponding *s* values for a hemisphere, a torus, an elastic tape, a flat punch, and a suction cup are 1/2, 1/3, 1/2, 1/4, and 0, respectively [30]. Equation (5) applies to ideal contact situations, where load is uniformly distributed across the adhesive pad, allowing all contact elements to be pulled off simultaneously and resulting in an adhesive force equal to the force of a single seta multiplied by the number of setae [31]. Beyond increasing the number of sub-contacts, the contact splitting model also suggests a built-in increase in contact area. Therefore, it is unclear from this model alone whether adhesion enhancement results from an increase in sub-contact numbers or simply an increase in contact area [32].

The effect of contact splitting on adhesion has been linked to the deformation behavior of individual fibers within the fiber array. Splitting a contact into finer sub-contacts enhances system compliance, increases tolerance to defects in individual contacts, and reduces the negative effects of surface roughness on adhesion and friction-based attachment [24,33,34,35,36,37,38]. However, excellent adaptability does not guarantee a perfect interfacial fit of the contact area [39]. Under non-ideal conditions, adhesion strength in arrays can be significantly lower than the sum of all individual fibrils’ adhesion [40]. For example, Kim and Varenberg [24] measured the mean pull-off forces and static friction forces of original and split wall-shaped adhesive microstructures against different rough surfaces under a constant normal load of 20 mN. We extracted and analyzed data from the experimental result figures in [24] to quantify the effects of contact splitting on pull-off force and static friction force, as presented in Table 1, where *R_q_* represents the root-mean-square deviation calculated from the filtered roughness profiles. The data indicate that splitting the microstructure enhances adhesion and friction across all surfaces, with the greatest effectiveness observed at intermediate roughness levels (*R_q_* = 0.16–0.22 μm).

Recently, Hu et al. [41] revisited the contact splitting principle through numerical modeling of adhesive contact between a fractal rough surface and either a non-fibrillar or fibrillar surface. Their results show that highly split structures are essential for strong adhesion on highly rough surfaces but may slightly reduce adhesion on smooth surfaces due to areal loss in the splitting process.

In artificial fibrillar adhesive synthesis, the progressive miniaturization of contact tips is limited by fiber self-adhesion [42,43], resulting from the interplay between adhesion forces, mechanical contact interactions, and structural resistance to axial, shear, and bending deformation [44]. However, real gecko setae arrays exhibit far less susceptibility to self-adhesion. One reason may be that setae tips have a sophisticated, non-uniformly distributed three-dimensional structure not yet replicated in artificial systems [45,46,47]. For slanted micropillars, the tilt angle affects length, compliance, and array density. At lower tilt angles, the benefit of reduced deflection for self-adhesion is outweighed by the increased bending rigidity with decreased tilt [48]. Consequently, critical length and center-to-center distances are required for micropillar arrays at a given tilt angle. For frictional interactions between micropatterned surfaces and soft substrates, such as deformable tissue, array density directly influences substrate deformation between neighboring pillars at a given indentation depth, resulting in increased lateral contact necessary for enhanced shear response [49,50].

Contact splitting promotes effective load distribution and adaptability in adhesive systems. To further optimize adhesion strength and controllability, it is essential to analyze crack initiation and propagation mechanisms at the interface using fracture mechanics principles. This approach provides insights into failure modes and detachment processes, enabling more precise control over adhesion performance.

### 2.2. Fracture Mechanics of the Interface Between Microfibrils and Substrate

#### 2.2.1. Total Energy of System and Fiber Deformation Equations

Fracture mechanics offers a framework to analyze crack initiation and propagation at the interface between the microfibrils and a substrate. For a fibril array with a deformable backing layer in contact with a rigid substrate, the system’s total energy can be expressed as follows:

(7) $$\Pi =U_{fibril\;array}+U_{backing}-W_{ext}-\Delta \gamma A$$

where $U_{fibril\;array}$ represents the strain energy within the fibril array, accounting for the combined deformation of all fibrils, including both their individual bending, stretching, and compression, as well as interactions among them. $U_{backing}$ denotes the strain energy within the deformable backing layer, which affects load sharing and compliance across the adhesive system in response to external loads. $W_{ext}$ is the work done by external forces, and ΔγA is the adhesion energy at the interface between the fibril array and the substrate.

The Principle of Stationary Potential Energy is expressed as

(8) δΠ=0

where δ is the variational operator. By applying the necessary variations, differential equations can be derived that accurately capture the collective behavior of the fibril array, with boundary conditions that reflect the interactions between the array, backing layer, and rigid substrate. For a fiber perfectly bonded to a rigid substrate with a contact area *A* and without a deformable backing, the resulting differential equation is given by

(9) $$EI\frac{d^{4}u}{dx^{4}}-\frac{d}{dx}\left(EA\frac{du}{dx}\right)=F(x)$$

where *u*(*x*) represents the displacement along the fiber length, *EI* is the flexural rigidity, *EA* is the axial stiffness, and *F*(*x*) represents the distribution of the applied external force along the fiber length. During pull-off or peeling, the external force will cause bending and axial deformation in the fiber, leading to stress concentration and displacement at the interface.

In a fibril array, each fiber is subjected not only to external forces but also to the elastic coupling effects of neighboring fibers, which influence its deformation behavior. The deformation of each fiber in the array impacts the overall stress and displacement distribution. For simplicity, studies on fibril arrays often model deformation using coupled differential equations as follows:

(10) $$EI\frac{d^{4}u_{i}}{dx^{4}}-\frac{d}{dx}\left(EA\frac{du_{i}}{dx}\right)=F_{i}(x)+\sum_{j\neq i}\alpha_{ij}(u_{j}-u_{i})$$

where $\alpha_{ij}$ is the coupling coefficient between fibers, indicating the strength of elastic interaction among them. By considering the appropriate boundary conditions, the resulting stress and displacement fields can be determined.

#### 2.2.2. Stress Intensity Factor in Microfibril Arrays and Effective Energy Release Rate

During fiber detachment from the substrate, crack progression is guided by a Griffith-type criterion. Here, the contact area, *A*, reduces as detachment progresses, and the energy release rate, *G*, is defined as

(11) $$G=\frac{\partial U_{elastic}}{\partial A}+\frac{\partial W_{ext}}{\partial A}$$

Each micro-contact has an associated local energy release rate, which is governed by its stress intensity factor. The stress intensity factor measures the stress intensity near a crack tip, which is essential for predicting interfacial behavior. In fibrillar systems, the stress intensity factors for modes I (opening) and II (shearing) can be expressed as

(12) $$K_{I}=\underset{r\to 0}{\lim}\sqrt{2\pi r}\sigma_{normal}(r,\theta ),\;K_{II}=\underset{r\to 0}{\lim}\sqrt{2\pi r}\tau (r,\theta )$$

where $\sigma_{normal}(r,\theta )$ and τ(r,θ) are the normal and shear stresses near the crack tip. In microfibril arrays, each fiber’s behavior is influenced by neighboring fibers, leading to complex stress distributions within the array, represented as

(13) $$K_{I}^{eff}=\sum_{i,j}\alpha_{ij}K_{I}^{\left(j\right)},\;K_{II}^{eff}=\sum_{i,j}\alpha_{ij}K_{II}^{\left(j\right)}$$

This interaction results in an effective energy release rate $G_{eff}$ for the array, which considers fiber interactions and system compliance.

(14) $$G_{eff}=\frac{{\left(K_{I}^{eff}\right)}^{2}+{\left(K_{II}^{eff}\right)}^{2}}{E^{*}}$$

where $E^{*}$ is the effective modulus. According to the *Griffith criterion*, crack propagation initiates when $G_{eff}\geq G_{C}$, controlling both adhesion and detachment behaviors.

#### 2.2.3. Critical Force Scaling and Predicting Adhesive Behaviors

From fracture mechanics, the critical force, *F_C_*, at which the interface undergoes unstable separation, scales as [51]

(15) $$F_{C}∼\sqrt{G_{C}}\sqrt{\frac{A}{C}}$$

where *C* is the system compliance in the loading direction, and *G_C_* is the critical strain energy release rate, which accounts for various dissipative processes, making it broadly applicable. This scaling relationship suggests that the design of reversible adhesive should balance maximizing true contact area with minimizing loading compliance. Equation (15) highlights that *G_C_* is a key control parameter for switchable adhesives and can be tuned by interfacial chemical or physical modifications [52,53,54,55]. Among these, physical bonds are commonly used to modulate *Gc* because they are easier to manipulate [13]. As Equation (15) suggests, effective adhesive designs minimize compliance to maximize force capacity [56]. In other words, materials with lower Young’s moduli are more prone to deformation and concentrated stresses [57], whereas stiffer adhesives resist contamination and facilitate self-cleaning [58].

While Equation (15) helps analyze control parameters in switchable adhesive design, a complete fracture mechanics analysis is needed to accurately predict adhesive behavior [59,60]. This analysis requires calculating the energy release rate at each contact point to evaluate potential detachment or failure. To predict the adhesive behavior of microfibrils, solving the associated coupled, non-linear, partial differential equations is essential to obtain solutions for this complex problem and the corresponding energy release rate. Over the past five years, several studies have employed different approaches to investigate attachment and detachment mechanisms in fibrillar adhesive systems, addressing various factors influencing these processes [61,62,63,64,65,66,67,68]. While traditional fracture mechanics provides valuable insights into interfacial adhesion, additional modeling approaches have been developed to account for factors such as load-sharing efficiency in micropatterned adhesive arrays. Booth and colleagues [40,69,70] developed a statistical model informed by experimental observations to describe the distribution of local adhesive strength and the resulting performance of a micropatterned adhesive. Their model, grounded in adhesion tests and in situ contact visualization, demonstrates that Weibull statistics can characterize strength distribution. In addition, several studies have employed a cohesive zone model to describe interfacial adhesion [71,72,73,74].

Clearly, the mechanisms governing adhesion involve both surface interactions and interfacial crack propagation. These principles provide a foundation for understanding how energy distribution, contact geometry, and material properties influence adhesion and detachment. However, predicting adhesion behavior in dynamic or variable environments often requires insights beyond basic contact and fracture mechanics. In such cases, coupled adhesion and friction mechanisms become crucial, as they integrate shear forces with adhesive interactions, to explain unique attachment and detachment behaviors. This coupling between shear and adhesion offers critical insights into the stability and adaptability of fibrillar adhesive systems under sliding or loading conditions, explaining how these systems achieve high adhesion strength while allowing for controlled detachment.

### 2.3. Coupled Adhesion and Friction

Autumn et al. [75] observed a remarkable tribological response in gecko setal arrays by measuring detachment angles of isolated setal arrays and live gecko toes. They proposed a phenomenological model called “frictional adhesion”, in which the adhesive force $F_{⊥}$ along the adhesive direction is limited by both the shear force $F_{∥}$ and a critical detachment angle, α*.

(16) $$F_{∥}\geq -\frac{F_{⊥}}{\tan\alpha^{*}}$$

The frictional adhesion model explains the low detachment forces observed in climbing geckos without requiring toe peeling. It enables precise control of adhesion through shear force, making adhesion “shear-sensitive” or directional. Shear sensitivity arises from changes in contact area and adhesive strength, which in turn depend on non-adhesive default positions, peeling mechanics, pad sliding, and the controlled storage and release of elastic strain energy [76].

A distinctive feature of natural fibrillar systems is the strong coupling between shear and adhesion [77]. With the addition of shear load, Coulomb’s law dictates that the friction force for each contact can be represented as

(17) $$F_{f}=\tau_{0}A_{real}+\mu P$$

where $\tau_{0}$ is the critical shear stress. For low normal loads, *P*, the adhesion-controlled friction (first term) dominates, while at high normal loads, pressure-controlled friction (second term) prevails. The influence of both terms depends on the specific application, particularly the smoothness, compliance, and adhesion of the contacting surfaces, as demonstrated by experimental studies [78,79,80] and supported by theoretical analyses [81,82]. In both biological and bio-inspired adhesive systems, sliding friction can be effectively described by continuum contact models that incorporate coupled adhesion and friction [83]. Within the same macroscopic contact area, local contact stresses in gecko toes can vary between tension and compression [84], making coupled adhesion–friction models well suited for the computational analysis of normal and tangential peeling behavior in gecko spatula [85,86].

Furthermore, understanding frictional behavior under zero or negative (tensile) contact pressures is beneficial for engineering applications like wall-climbing robots and human climbing. Simulations by Mergel et al. [87] suggest that a shear-induced reduction in contact area, discussed in [83,88,89,90,91,92,93,94,95,96], can occur even without adhesion and in compressible materials. At the material level, factors such as work of separation, elastic modulus, and imperfections in the sample influence the friction profile when the adhesive is under shear loading [97].

Though the fundamental models for the gecko adhesive system are highly simplified, they allow the rapid exploration of design principles for bio-inspired adhesives [98]. Over the past two decades, experimental studies and theoretical models have expanded our understanding of the mechanics of wedge-shaped, mushroom-shaped, and spade-shaped fibers, as well as arrays of these structures (e.g., Refs. [77,99,100,101,102,103,104,105,106]). Advances in computational tools and experimental techniques now make it possible to design, manufacture, and evaluate novel bio-inspired adhesives within the constraints of available materials and manufacturing methods.

In practical applications, bio-inspired gecko adhesive systems typically consist of a substrate and an array of fibrils connected to a backing layer. Effective adhesion strength varies with contact forces, which in turn are influenced by factors such as substrate roughness, mechanical properties of the fibers, backing layer stiffness, and environmental conditions. As previously noted, debonding occurs when cracks initiate and propagate at the interface between microfibrils and the substrate. The propensity for crack propagation is determined by the local stress state at the crack tip for a given loading condition. In the following section, we will discuss recent studies examining how these factors affect interfacial stress distribution and adhesion.

## 3. Effective Adhesion Strength of Artificial Fibrillar Adhesives

### 3.1. Contact Geometry and Stiffness of Microfibrils and Backing Layer

**Figure 2:**
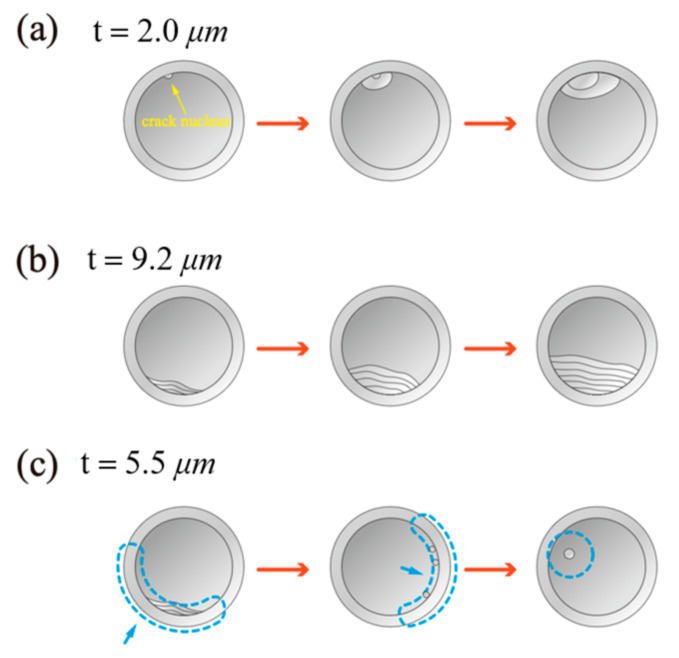
Three characteristic modes of detachment of the mushroom-shaped fibril with various cap thicknesses (redrawn based on [107]). (**a**) Interior-crack mode. (**b**) Edge-crack mode. (**c**) Mixed interior/edge-crack mode.

When a fiber adheres to a substrate, the contacting surfaces rarely match perfectly, causing an interfacial crack to initiate at the contact corner and propagate toward the fibril center. Adhesion experiments and in situ observations of mushroom-shaped microfibrils [107] identified three crack modes: interior-crack mode, edge-crack mode, and mixed interior/edge-crack mode, each related to the thickness of the cap, as shown in Figure 2. As a result, the stress singularity at the contact corner drives unstable crack growth, ultimately decreasing adhesion strength [61]. To better understand and predict this behavior at the fiber/substrate interface, numerous studies have focused on the mechanics of crack initiation and propagation and how various factors influence these processes [108,109,110,111,112]. For example, Labonte and Federle [31] examined how contact geometry and detachment modes affect the scaling of attachment forces for adhesive pads. They showed that uniform stress distribution (area scaling) results in strong adhesion, while stress concentrations (length scaling) facilitate detachment. Rapidly controllable adhesives likely require the ability to switch between area and length scaling, with shear forces playing a crucial role. Additionally, tensile forces can reduce stress concentrations, thereby impacting adhesion scaling.

Recent research has explored ways to mitigate stress concentrations and optimize interfacial stress distribution [113,114,115,116,117,118]. For example, Benvidi and Bacca [115] used linear elastic fracture mechanics (LEFM) to analyze stress on uncracked interfaces with varying adhesive geometries, specifically focusing on the thickness of the soft layer to identify regions prone to stress concentration. They found that as the thickness increases, the stress concentration at the center of the interface decreases. Conversely, when thickness falls below a certain threshold, a further reduction in thickness leads to a more uniform stress distribution. They observed two primary detachment mechanisms: (i) center crack propagation, which provides stable crack growth and toughens the interface when the soft tip is sufficiently thin, and (ii) edge crack propagation. Samri et al. [116,117] compared experimental results with 3D numerical simulations to assess the influence of microstructure on contact stiffness and effective modulus. Additionally, Tarpey and Ronan [118] conducted numerical studies on how fibril tip shape and elastic mismatch at the fibril–substrate interface impact adhesion.

### 3.2. Substrate Roughness

Synthetic fibrillar adhesives can now achieve or even surpass the adhesion strength of gecko pads on smooth surfaces. However, the adhesion mechanics of these systems are highly dependent on contact area, relying on van der Waals forces. While smooth, clean surfaces have been the focus of much gecko adhesion research, they are actually among the least challenging surfaces for geckos to adhere to [119]. In practical applications, however, most substrates exhibit surface roughness across a wide range of scales, from sub-nanometer to millimeter or even centimeter levels. When synthetic fiber adhesives make contact with rough surfaces, the effective adhesion strength decreases due to a reduction in the real contact area caused by local bending and buckling of the fibrils [1,120]. Adhesion strength drops significantly when surface roughness exceeds the size and spacing of the adhesive fibrillar structures [121,122]. Furthermore, increasing substrate roughness reduces pull-off force at smaller orientation angles [123,124]. While substrate roughness is often recognized as a cause of partial contact [122,125], other substrate properties, such as patterning, curvature, and compliance, also influence the peeling behavior of adhesives [126,127,128,129]. Recent studies have shown a non-linear relationship between adhesion and roughness [130,131]. Although understanding how roughness impacts adhesion is crucial for determining the effective adhesion strength, many questions remain unanswered, fueling ongoing research in this area.

### 3.3. Environmental Factors and Pull-Off Dynamics

The effects of temperature and humidity on adhesion have been investigated experimentally, showing that these factors interact in complex ways that can result in up to a two-fold difference in adhesion strength [132]. A theoretical study later explored the coupled effects of temperature and humidity, finding that adhesion decreases with rising temperature if humidity is uncontrolled. However, if humidity remains constant, the peel-off force is insensitive to the temperature and remains almost constant [133]. The precise mechanisms driving the combined effects of humidity and temperature on gecko adhesion remain unclear. Independently, humidity’s influence on adhesion is better understood; it increases adhesion via capillary forces and by softening materials, which enhances contact area [134]. For a detailed review of the role of water and humidity in both gecko and synthetic adhesion, see Ref. [135]. Recent studies have increasingly focused on the mechanics of biological and synthetic adhesives under varying environmental conditions, with new insights contributing to improved performance in humid or wet conditions [136,137,138,139,140]. Beyond environmental factors, pull-off dynamics also play a crucial role in adhesion mechanics, particularly influencing the mode of crack propagation [141,142,143,144]. Studies on rate-dependent adhesion in fibrillar adhesives show that the relationship between pull-off force and velocity follows a power law at high retraction velocities for both center and edge crack propagation modes [143]. This behavior results in stress redistribution that counteracts the effects of retraction velocity [145].

Advances in understanding the mechanics of attachment and detachment in fibrillar adhesive systems have deepened insights into gecko-inspired adhesive mechanisms. Table 2 summarizes recent approaches and key focus areas in the interface mechanics of fibrillar adhesives, highlighting essential methods and insights that inform the design and optimization of these biomimetic systems.

**Table 2:** Recent advances in mechanics and statistical and data-driven approaches for dry adhesives.

Research Approaches	Key Methods/Models	Key Research Focus	System Studied	Example Applications
Fracture Mechanics and Numerical Simulations	Linear Elastic Fracture Mechanics (LEFM) [60,61,99,103,115,118,146,147]	Stress distribution, crack initiation and propagation	Single fibril and arrays	Predicting detachment in adhesive systems
	Cohesive Zone Modeling [68,71,72,73,74,107]	Interfacial energy dissipation and failure	Fibril–substrate interface	Enhanced control over detachment mechanics
	Hybrid Dynamic Fracture Mechanics [101,141,142,143,144,145]	Dynamic adhesion characteristics and rate-dependent work of adhesion	Single-fibril, micropatterned surfaces	Rate effects in synthetic adhesive structures
Contact Mechanics	Contact Splitting [24,41], Peeling and Pull- Off [44,63,96,131], Equal Load Sharing [114,146], Traction–Separation Relation [50,100,102]	Load distribution and contact area scaling, enhancing adhesion on rough surfaces, backing-layer	Single fibril or arrays	High-strength adhesion with load-sharing, roughness effects
Elasticity Models	Spring Models [67,128]	Compliance and load-sharing	Single fibril or arrays	Designing optimal adhesion under loading
Statistical Models	Weibull Statistics, Probabilistic Models [40,69,70]	Variability in adhesion due to defects	Fibril array	Robust adhesion performance with defects
Coupled Adhesion and Friction	Frictional Adhesion Modeling [83,85,86,87]	Effects of shear force on adhesion	Arrays	Applications in robotic grippers
Buckling and Bending Analysis	Analytical or Numerical [48,148]	Stability under compressive loads, self-adhesion	Single fibril or array	Enhanced adaptability to uneven surfaces
Data-Driven Modeling	Supervised Machine Learning [65,66,117,149]	Prediction of adhesion strength, pattern recognition	Micropatterned surfaces	Performance optimization in adhesion design

Integrating these progresses into the design of bio-inspired adhesives opens pathways for enhanced functionality across diverse and challenging surface conditions. The following section examines specific case studies that illustrate how these advancements in adhesive mechanics can be strategically applied to optimize the adhesion properties of fibrillar adhesives, enabling promising applications in robotics, medical devices, and industrial automation.

## 4. Enhancing or Tuning Adhesion: Case Studies

One efficient strategy to enhance adhesion strength is reducing interfacial stress singularities. A wide array of studies has aimed at optimizing load distribution, for instance, by tuning the properties of backing layers, adjusting fibril stiffness based on location [113,146,150], or applying targeted displacements to the tip layer [151]. Achieving tunable adhesion in fibrillar adhesives is also essential for practical applications. Recent advancements involve external stimuli, such as mechanical forces and thermal, electric, or magnetic fields, to control the extent of true surface contact or enable directed shear adhesion, which are instrumental in developing gripping systems. This section presents case studies on enhancing adhesion and achieving reversible control.

### 4.1. Innovative Design and Optimization of Fibrillar Adhesives

**Figure 3:**
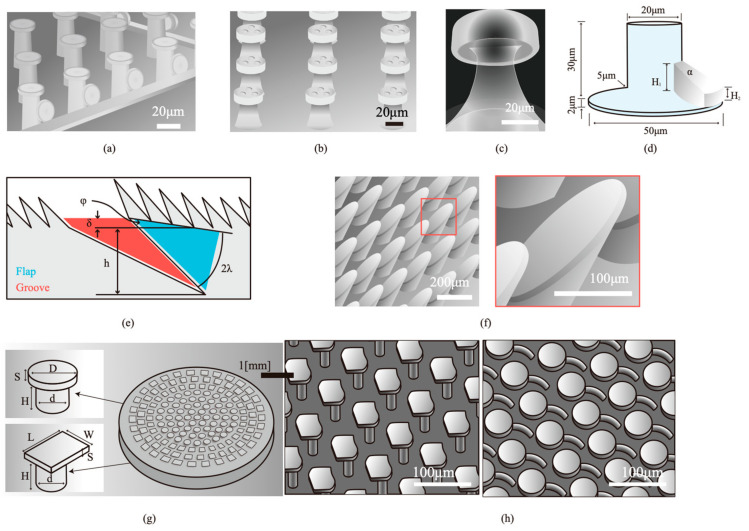
Innovative multifunctional mushroom-shaped fibrils and directional adhesives (redrawn based on [153,154,155,156,157,159,161]).

The contact shape plays a significant role in determining the adhesive properties of single fibers and fiber arrays. Mushroom-shaped tips with larger dimensions demonstrate the highest pull-off forces due to increased stress at the contact center and reduced edge stress concentrations [107,108]. Recent research has explored adding multifunctionality to mushroom-shaped fibrils and designing innovative directional adhesives. For example, Fe_3_O_4_/PDMS composites with robust self-cleaning capabilities have been developed [152]. In comparison, liquid super-repellent bio-inspired fibrillar adhesive surfaces exhibit strong adhesion in both underwater and dry conditions. Examples include a springtail–gecko-inspired adhesive structure array featuring side-surface and top-surface liquid super-repellency with strong reversible adhesion (Figure 3a); an array of octopus–gecko-inspired adhesive structures exhibiting high adhesion in both underwater and dry conditions (Figure 3b) [153]; a liquid-superrepellent bio-inspired fibrillar adhesive surface (Figure 3c) [154]; and a mushroom-shaped structure with an integrated trapezoidal prism, which provides switchability while maintaining adhesive performance (Figure 3d) [155].

In contrast, gecko-inspired anisotropic microstructures (e.g., tilted or asymmetric fibers) offer controlled adhesion properties by adjusting the shear direction of the contact surface. Figure 3e shows a microstructured adhesive with continuously varying features, which achieves highly asymmetric friction with a friction ratio of 100:1 [156]. For applications in space debris capture, Busche et al. [157] developed an adaptable 3D printing process based on two-photon polymerization with a multi-step molding routine, which allows precise replication of surfaces with micropillar structures and controllable adhesion behavior (see Figure 3f). Some designs achieve effective anisotropic adhesion by introducing asymmetry in mushroom-shaped fibrils [158,159,160,161,162]. Examples include a combination of micro-mushroom and micro-spatulae textures (Figure 3g) [159] and microfiber structures with off-center caps for controlled adhesion (Figure 3h) [161].

Additionally, innovative methods for achieving strong adhesion to both smooth and rough surfaces have recently focused on material and geometric optimization. For instance, applying an extremely soft, thin terminal layer of pressure-sensitive adhesive decorated with elastic mushroom microfibers has resulted in high adhesion strengths of up to 300 kPa [163]. Another approach involves embedding polydimethylsiloxane (PDMS) micropillars with a modulus gradient, created by dispersing calcium carbonate nanoparticles in the micropillar stalks, producing a stiff tip with a soft root for effective adhesion [164,165,166]. Simaite et al. enhanced gecko-inspired adhesive bonding by adding crosslinkers and catalysts to silicone elastomers, forming hybrid gecko-like adhesives [53]. Additionally, integrating stiff fiber reinforcement with a compressible backing layer enables conformation and strong attachment to non-flat substrates [167]. Tong et al. [147] applied topology optimization techniques to optimize stiffness distribution within a multimaterial adhesive backing layer, effectively reducing stress concentrations at specific locations, such as the crack tip. Pande and Turner [168] further advanced adhesive designs by employing a data-efficient, gradient-based geometric optimization scheme to develop adhesive pillars with near-ideal stress distributions.

**Figure 4:**
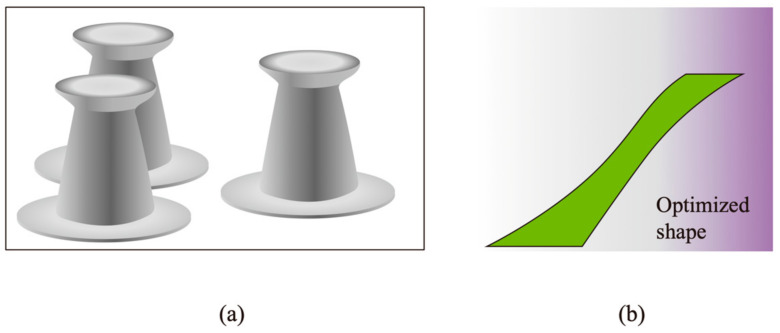
(**a**) Optimal mushroom-shaped tips of fibril. Scale bar: 50 μm. (**b**) Optimized directional adhesive pillar shape with excellent adhesive strength and controllability. (Redrawn based on refs. [171,173]).

Recent research has introduced 3D printing techniques and machine learning-based optimization methods for developing bio-inspired fibrillar adhesives [149,169,170,171,172,173,174]. For example, a machine learning-based strategy identified optimal designs for adhesive composite pillars with stiff cores, achieving critical normal detachment forces nearly 11 times higher than those of homogenous pillars [169]. Shi et al. [170] utilized high-resolution 3D printing techniques to fabricate micropillars with asymmetrically tilted orientations, achieving anisotropic adhesion with a two-fold increase in strength along the gripping direction compared to the releasing direction. Their work highlights the role of precision microfabrication in controlling adhesion behavior through structural anisotropy. Another study employed machine learning to improve mushroom-shaped tips (Figure 4a), with optimal designs that enhanced dry adhesion performance on smooth surfaces by up to 77% over previous designs [171].

Kim et al. [172] used deep learning for nonparametric shape optimization, achieving high adhesion strength by maximizing the uniformity of interfacial stress distribution. Additionally, multimaterial 3D printing has further expanded the design possibilities for fibrillar adhesives. As demonstrated in [173], Stratasys PolyJet printing enables the fabrication of fibrillar structures with controlled stiffness variations by combining high-stiffness (2500 MPa) and soft (0.886 MPa) materials, allowing for tunable adhesion performance. Their systematically optimized directional adhesive pillar shapes (Figure 4b) demonstrated excellent adhesion strength and control and were successfully fabricated with multimaterial 3D printing technology [173].

To further highlight recent advancements in fibrillar adhesive design and optimization, Table 3 provides a summary of key innovations and approaches within this evolving field.

**Table 3:** Recent innovative design and optimization approaches for fibrillar adhesives.

Design Strategy	Applications	Advantages/Innovations
Dry-adhesive microstructure for rough surfaces [7]	Material handling on Mars	392.94× increase in pull-off stress; enables low-energy handling of rough, additively manufactured surfaces
Springtail-, gecko-, and octopus-inspired 3D microstructures [153]	Medical devices, robotics, wearable adhesives	Strong reversible adhesion; super-repellency on wet and dry surfaces; versatile for synthetic/biological surfaces
Mushroom-shaped fibrillar arrays with double re-entrant tips [154]	Robotics, medical devices, underwater adhesives	Retains adhesion in presence of water, oil, and other liquids; robust, stretchable, and highly deformable
Trapezoidal-prism + mushroom-shaped microstructure [155]	Pick-and-place for microelectronics, transfer printing	Strong adhesion (87.8 kPa); low detachment strength (<0.07 kPa); works in dry and wet conditions
Gecko-inspired adhesive with spatial variation [156]	Robotics, climbing devices	100× stronger adhesion in preferred direction; enhances control and mimics natural adhesive asymmetry
Anisotropic dry-adhesive microstructures produced via two-photon polymerization [157]	Space debris capture, high-anisotropy applications	High anisotropic adhesion factor (7.52:1); strong adhesion (up to 1105.29 mN/cm^2^); suitable for mass production
Combined microfibril textures (micro-spatulae and micro-mushroom) [159]	Robotics, adaptive gripping systems	Optimized adhesion and friction under varying loads and velocities; adaptable to environmental conditions
Off-center spatula-shaped microfiber caps [161]	Pick-and-place, robotics	3–5× reduction in adhesion for easy detachment; 3× increase in shear with lateral drag; versatile directional control
Mushroom-like PDMS microline arrays with directional patterns [162]	Silicon wafer transportation	High shear adhesion in parallel direction; low peeling force, enabling easy detachment and directional control
Elastomeric mushroom-shaped microfibers with PSA layer [163]	Wearable medical devices, transfer printing, robotic manipulation	High adhesion strength (300 kPa); 35× durability improvement on smooth surfaces
T-shaped PDMS micropillars with modulus gradient [164]	Robotics, gripping systems	4.6× increase in adhesion; 2.4× increase in friction compared to pure PDMS arrays
Fiber-reinforced adhesive with bio-inspired soft backings [167]	Soft robotic grippers, curved surfaces	Enhanced conformability and friction on curved substrates; scalable contact area inversely with backing stiffness
Mushroom-like adhesive pillars with optimized geometry [168]	Robotic grasping, microtransfer printing	2× adhesion enhancement; reduced edge stress concentration; crack initiation shifted to pillar center
Machine learning-optimized composite pillars [169]	Robotics, adhesive interfaces	11× increase in detachment force; high adhesion under varied loading conditions; 1.7× improvement over rectangular core design
Gecko-inspired micropillars with asymmetrical tilt [170]	Robotics, gripping systems	4× stronger adhesion than plain surface; 2× stronger in gripping direction than release direction
Machine learning-optimized gecko-inspired fibrils [171]	Robotics, medical devices	77% improvement in adhesion; sensitivity to fibril shape and deformation considered
Free-form optimized adhesive pillar shapes [172]	Robotics, adhesive interfaces	Improved uniformity in stress distribution without stress peaks
Directional adhesive pillars with anisotropic properties [173]	Wall-climbing robots, grippers	Superior directionality and adhesion strength
In-plane combination of micropillars with different aspect ratios [175]	Space capture and docking	Maintains 85% adhesion after large deformation; resists overload-induced adhesion failure; adaptable to dynamic capture

### 4.2. Strategies of Reversibly Controlled Adhesion and Latest Advances

The flexible control of dry adhesives has gained significant interest in fields such as automation and robotics, where dry-adhesive grasping systems present promising applications. The design of such systems hinges on understanding how adhesion strength can be altered, identifying design parameters linked to detachment mechanisms, and leveraging these factors for specific applications. In fibrillar adhesive systems, adhesion control strategies include adjusting the true surface contact area by altering stiffness or stress distribution at the interface, as well as modulating pulling angles and shear forces to achieve directed shear adhesion. Here, we review recent advances in reversible adhesion control strategies.

#### 4.2.1. Controlling the Amount of True Surface Contact

According to fracture mechanics principles, the force capacity *F_C_* of dry adhesives can be tuned by adjusting the reversible adhesive scaling parameter, $\sqrt{A/C}$. Flexible control over adhesion strength is thus achieved by manipulating the true surface contact area, either through shape changes or by adjusting the stiffness of the adhesive system [12,148,176,177].

**Figure 5:**
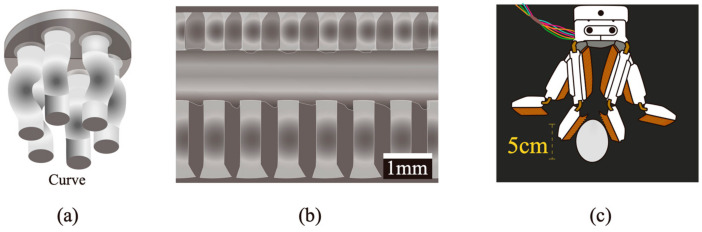
Examples of switching adhesion exploiting buckling. (**a**) The “curve” fibrils [178]. (**b**) Switchable double-sided micropatterned adhesives [179]. (**c**) Manipulator integrated with buckling ribs [182]. (Redrawn based on [178,179,182]).

One straightforward approach to modulating contact is through elastic buckling, where fibrils deform or buckle under pressure, reducing contact area and, thus, adhesion strength. Recently, elastic buckling has been used to design switchable adhesive structures and robotic manipulators [178,179,180,181,182,183,184]. For example, the “curve” fibrils design (Figure 5a) exhibited a high switching ratio of around 20 due to irreversible sliding of fibrils during compression [178]. By tuning the dimensions of each face of a double-sided dry adhesive pad (Figure 5b), the critical load for buckling and the preferred face of detachment can be selectively adjusted [179]. Figure 5c shows a robotic manipulator that uses buckling ribs to enable shear load sharing and normal compliance [182].

**Figure 6:**
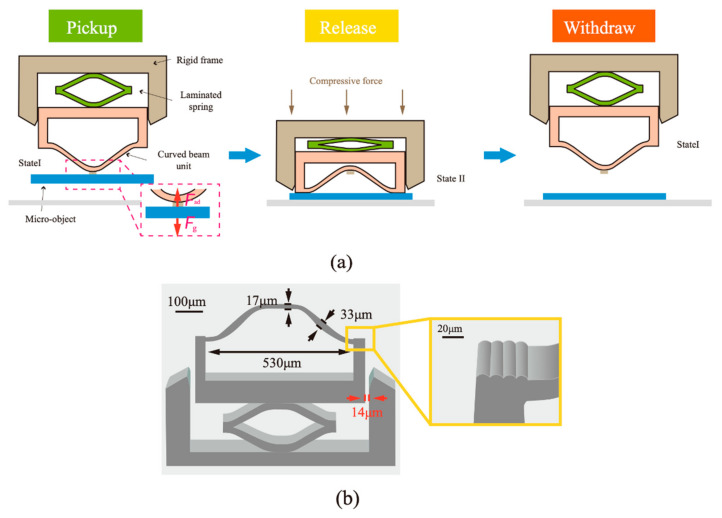
(**a**) Schematic demonstrating the switching principle using a snap-through metastructure. (**b**) Miniaturized device. (Redrawn based on [185]).

Detaching superlight objects without damaging them presents a unique challenge, particularly in micro-robotic handling. To address this, adhesive devices need to be designed that can generate high adhesion forces yet allow easy release. Unlike methods that alter the adhesive state of the interface, Zhang et al. [185] applied a mechanical metastructure concept using a snap-through instability in a curved beam to grip and release microscopic objects. Here, compression-induced snap-through causes a switch in the contact area between the curved beam and the target object (Figure 6a). For effective detachment, the ends of the curved beam were modified with low-adhesive wavy surfaces (Figure 6b). This approach achieves a switching ratio exceeding 10^4^—three orders of magnitude higher than typical buckling-induced release methods using micropillars [182,186].

**Figure 7:**
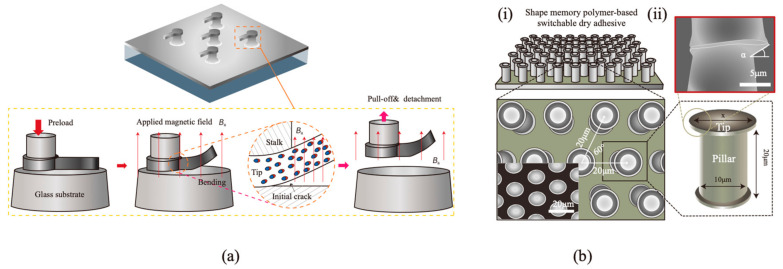
Examples of switching adhesion by modifying the tip shape of the fiber. (**a**) A smart adhesive to achieve dynamically tunable adhesion [187]. (**b**) Structures of the SSA: (**i**) design of the SSA and their layouts and (**ii**) SSA’s pillar and tip dimensions and SEM image of slanted SSA tip [188]. (Redrawn based on [187,188]).

At the fiber level, modifying the tip shape can also influence the contact area. For example, a mushroom-shaped adhesive with a magnetized tip can rapidly alter its morphology through magnetic actuation (see Figure 7a). When an external magnetic field is applied, the tip bends, initiating a crack at the edge of the contact area. The crack propagation transitions the adhesion from strong to weak, enabling immediate object release [187].

Another innovative approach involves a shape memory polymer (SMP)-based switchable dry adhesive (SSA) (see Figure 7b). This adhesive exhibits excellent adaptability across surface roughness ranges from 0.8 to 45 μm and achieves a switching ratio of about 89. Upon thermal stimulation, the SSA transforms from conformal to line contact, significantly reducing the contact area. This makes it ideal for pick-and-place systems designed to handle ultra-thin, lightweight objects that are prone to damage under slight external forces [188].

**Figure 8:**
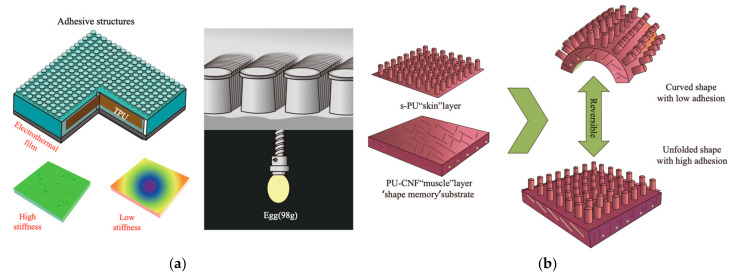
Examples of switching adhesion by tuning structural stiffness. (**a**) Hierarchal adhesive structure [190]. (**b**) Switchable superhydrophobic shape-memory adhesive film [192]. (Redrawn based on [190,192]).

At the material level, recent studies have focused on enhancing adhesion control by adjusting structural stiffness [189,190,191,192,193,194]. For instance, Li et al. [190] developed a hierarchical adhesive structure composed of a top mushroom-shaped layer, a stiffness-modulating thermoplastic polyurethane (middle layer), and an electrothermal film (bottom layer) (Figure 8a). By applying voltage to control stiffness, this design achieves adhesion forces that are 10 to 100 times stronger than conventional adhesives, supporting both attachment and detachment functions. Another innovative example involves a shape-memory superhydrophobic film with switchable adhesion based on bending and unfolding properties [193]. This film features a layer of pillar-structured s-PU adhered to a water-responsive shape-memory PU-CNF substrate (see Figure 8). When exposed to water, only the underlying PU-CNF substrate is activated, leaving any microdroplets on the film unaffected. The shape-memory effect allows the film to exhibit different adhesive behaviors, which can be “memorized” without an external force, making it ideal for controlled droplet manipulation.

#### 4.2.2. Controlled Adhesion by Shear Force

Previous studies have demonstrated that the spatula-shaped tips on a gecko’s toes allow it to modulate adhesion and friction forces by laterally sliding and adjusting the pulling angle, enabling changes in adhesion over three orders of magnitude [195]. From an application perspective, reversible adhesion in asymmetric geometry or curvature fibrillar systems, similar to a gecko’s spatula-shaped adhesive pads, can be achieved either by adjusting peeling angles or by applying lateral shear forces. Recent research has also focused on developing directional adhesives with anisotropic adhesion properties to enable controllable adhesion, reusability, and high durability for various applications, including climbing robots [10,196], space debris removal [175], and soft grippers [197,198]. These efforts have explored different strategies for reversibly controlling adhesion.

**Figure 9:**
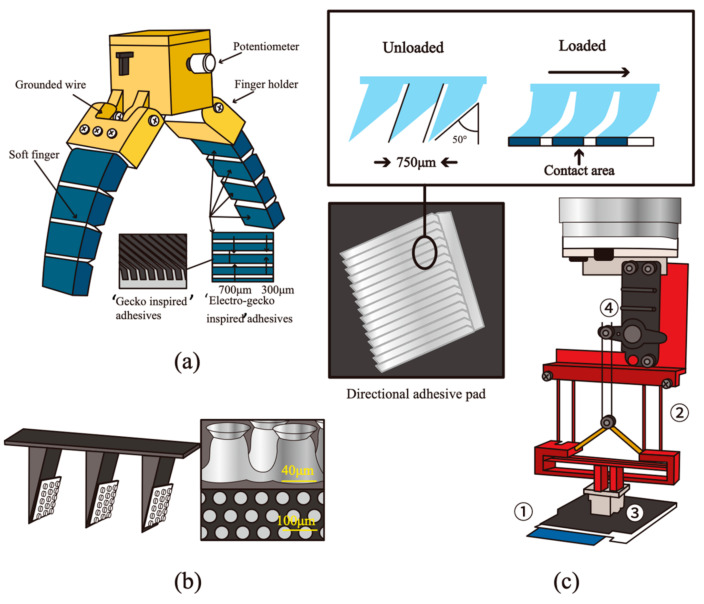
(**a**) Electrostatic/‘gecko-inspired’ adhesive soft robotic gripper [200]. (**b**) Hierarchically wedge-mushroom-shaped adhesive and magnified side view SEM on the wedge surface [201]. (**c**) A soft gripper, including (1) object, (2) compliant mechanism, (3) directional adhesive with curved shape, and (4) servo motor [202]. (Redrawn based on [200,201,202]).

To address the challenge of contamination, Alizadehyazdi et al. [54,199] investigated the influence of material properties such as the modulus of elasticity, work of separation, and adhesion energy on the shear stress and particle detachment capabilities of wedge-shaped directional microstructured adhesives. They used a piezoelectric element to remove dust from the adhesive surface and simultaneously tuned the adhesion strength, turning the adhesive “off” when needed, while also using the element as a force/contact sensor. In addition, electrostatic and gecko-inspired adhesives have been combined with soft grippers [200], enabling them to work across a broader range of materials, surface roughness, and irregular geometries (see Figure 9a). More recently, Shen et al. [201] developed a hierarchical adhesive comprising asymmetrical wedges with embedded NdFeB particles and mushroom-shaped fibrils on the sloping surface of the wedge (see Figure 9b). This switchable adhesion, controlled by a magnetic field, overcomes the limitations of shear-induced adhesion loss in normal force modes (e.g., loading, dragging, and pulling). The hierarchical adhesive also demonstrated excellent self-cleaning capabilities and the ability to release ultra-light objects. In another study, directional adhesive pads and a compliant gripper mechanism for soft grippers were fabricated using a 3D printing process. These were designed to grip a variety of flexible and flat objects, including films and flexible printed circuit boards (FPCBs) [202] (see Figure 9c).

The selected case studies illustrate how recent advancements in the mechanics of adhesion and detachment have been successfully applied to the design of bio-inspired adhesives. These examples demonstrate innovative approaches from recent years that leverage mechanical principles to enhance adhesion strength and enable controlled detachment, offering valuable insights into practical applications. For additional examples of innovative designs and applications, refer to Refs. [1,2,3,4,5,6,7,8,9,10,11,12,13,14,15,16,17]. Table 4 summarizes recent innovations in achieving reversible control of adhesion in fibrillar adhesive systems, highlighting key advancements and design strategies that enhance functionality and adaptability across various applications.

## 5. Discussion

The development of bio-inspired adhesives has made significant strides in recent years, particularly with advancements in mechanisms for controlling adhesion and detachment in fibrillar systems. These bio-inspired systems, drawing inspiration from nature, such as the adhesive mechanisms of geckos and other animals, have enabled new approaches to adhesion with high versatility, controllability, and reusability. By leveraging the principles of contact mechanics, hierarchical structuring, and responsive materials, researchers have designed adhesives that can adapt to different surface types, environmental conditions, and functional requirements.

### 5.1. Key Mechanisms and Innovations

Central to the success of bio-inspired adhesives is the design of structures that can exploit large contact areas without sacrificing the ability to detach when required. The introduction of hierarchical architectures, such as mushroom-shaped fibrils or asymmetric geometries, has been pivotal in enhancing the performance of these adhesives. These structures allow for optimized force distribution and more efficient adhesion, leading to improved grip on both smooth and rough surfaces. Additionally, innovations in tunable adhesives, such as those incorporating shape-memory polymers or electroactive materials, have opened new possibilities for dynamic adhesion that can be controlled remotely via external stimuli (e.g., temperature, electric field, or magnetic field).

Furthermore, advancements in 3D printing technologies have made it possible to create complex, multimaterial adhesive systems with unprecedented precision. These printing techniques allow for the design of adhesive structures with tailored properties, including variations in stiffness, roughness, and material composition, all of which can be fine-tuned to meet the needs of specific applications.

### 5.2. Challenges in Interface Mechanics

One of the most pressing challenges in the field of bio-inspired adhesives is the complexity of modeling and understanding the behavior of the contact interfaces between fibrils and substrates. Despite considerable progress in this area, existing models often struggle to capture the intricate interactions that occur at the micro- and nano-scales, particularly under varying load conditions. While contact mechanics theories provide valuable insights, the microscopic details of stress distribution, fracture behavior, and the effects of surface roughness remain difficult to predict with high accuracy. Multiscale modeling approaches, combining molecular dynamics, finite element analysis, and experimental data, could provide a more comprehensive understanding of these interactions. Additionally, novel experimental techniques, such as high-speed microscopy or synchrotron-based imaging, are needed to visualize and quantify the stress distribution and fracture behavior at the interface in real time.

Furthermore, the reliability of adhesive systems under real-world conditions—where factors like contamination, wear, and varying environmental factors come into play—remains an open question. While many studies focus on idealized conditions, real-world applications will require adhesives that maintain high performance over extended cycles of loading, unloading, and environmental exposure. As such, the development of more accurate and scalable models for adhesion mechanisms, especially under complex real-world loading conditions, remains a critical area for future research.

### 5.3. Precision, Repeatability, and Practical Applications

As bio-inspired adhesives move closer to real-world applications, one of the most critical challenges will be achieving precision in the control of adhesion properties. While tunable adhesion offers immense potential, ensuring that these systems can reliably and repeatedly switch adhesion on and off in a controlled manner is non-trivial. Many systems rely on external stimuli (e.g., electric fields, temperature gradients, or magnetic fields) to modulate adhesion, but the precision of this control—especially under varying operational conditions—remains a key hurdle.

In addition to precision, repeatability and durability are major concerns for any practical adhesive technology. Many existing adhesive systems exhibit degradation or loss of performance after repeated use, making long-term reliability a challenge for applications that require consistent performance over extended periods. The development of robust feedback mechanisms capable of monitoring and adjusting adhesion strength in real time could provide a solution to this challenge.

### 5.4. Expanding the Application Range

While the most prominent applications of bio-inspired adhesives have focused on robotics, aerospace, and soft grippers, the potential for these technologies to expand into new fields is vast. One particularly promising area is the development of adhesives for medical devices, particularly those used in minimally invasive surgeries or wearable health monitoring systems. In these applications, the adhesive must not only provide a secure grip but also be gentle enough to minimize damage to sensitive tissues or skin.

However, several technical challenges must be addressed before these adhesives can be widely adopted in biomedical applications. Ensuring strong yet reversible adhesion on dynamic, moisture-rich biological surfaces remains a significant hurdle. Additionally, bio-inspired adhesives for medical use must exhibit biocompatibility, long-term stability, and resistance to contamination, all while maintaining their functional properties over extended usage cycles. Addressing these challenges necessitates innovations in material selection, structural optimization, and adhesion mechanisms tailored for hydrated and dynamic biological environments.

## 6. Conclusions and Outlook

We have reviewed the state of the art in the interface mechanics of fibrillar adhesives and examined innovative design strategies for fibrillar structure optimization and reversible adhesion control. A summary of key innovations and approaches in this evolving field over the last five years is provided in tables for quick reference. Despite the tremendous recent progress surveyed in this area, many open questions and research opportunities remain. The following areas/problems can be explored by the dry adhesives research community from the mechanics perspective:

(1) Enhancing adhesion under dynamic and rate-dependent conditions—Investigating how adhesion strength and detachment behavior evolve under varying loading rates and dynamic environments using hybrid dynamic fracture mechanics and cohesive zone modeling.

(2) Optimizing adhesion on rough and uneven surfaces—Advancing mechanics-based strategies to optimize interfacial stress distribution and load-sharing mechanisms, particularly for micropatterned surfaces and fibrillar arrays, to achieve robust and high-strength adhesion in real-world applications.

From the reversible adhesion control perspective:

(1) Advancing rapid and reversible adhesion switching mechanisms—While various strategies such as shape-memory polymers, electrostatic tuning, and hierarchical structures have enabled adhesion switching, further research is needed to improve response time and energy efficiency in practical applications.

(2) Improving long-term durability—Ensuring that adhesives maintain performance over thousands of adhesion cycles without degradation, which requires advancements in contamination-resistant materials and optimized fibrillar architectures.

Addressing these challenges requires an interdisciplinary approach, integrating mechanics, materials science, computational modeling, and bio-inspired design. Future advancements in dry adhesives will not only enhance their practical performance but also expand their applicability to emerging fields such as space exploration, medical devices, and wearable technologies.

## Figures and Tables

**Table 1:** Effect of contact splitting on pull-off force and static friction force (data from [24]).

	Refrigerator	Table Desktop	Sputter Coater	0.3 μm FibrMet disc
*R_q_* (μm)	0.057	0.162	0.22	0.226
Pull-off force (mN) (Original/Split)	42/54.2	30/44.5	30.1/48.5	37/41.8
Static friction force (mN) (Original/Split)	360/398	280/374	250/308	335/310

**Table 4:** Recent innovative design for reversible control adhesion in fibrillar adhesive systems.

Design Strategy	Control Mechanism	Applications	Advantages/Innovations
Suction + gecko-inspired adhesion [3]	Suction for grip; nylon fabric with gentle airflow	Retail and warehouse robots	Lifts up to 2.3 kg; grips small items; conforms to irregular surfaces
Soft–hard–soft sandwiched composite for reversible adhesion [176]	Lateral shrinkage for uniform loading; stress concentration for easy detachment	Precision manufacturing, flexible electronics, climbing robots	Scalable adhesion (1.5 to 150 cm^2^); supports loads from 20 to 700 N; fast switching (~0.2 s); adhesion switching ratio of ~54
Hierarchical bionic toe (bio-toe) with elastic actuator and bionic lamellae [177]	Bi-directional pressure for adhesion/release and non-linear deformation for adaptability	Robot grippers, wall-climbing robots, space, defense	High adaptability and load capacity; 100% release success; 12× shear adhesion force-to-preload ratio; contact rate of 60% even with contact tilt
Directional buckling micropatterned adhesives [178]	Compressive overload for elastic buckling and controlled release	Pick-and-place systems, micro-assembly	High switching ratio (~20); precise release of objects <1 mm; enhanced placement accuracy
Double-sided micropatterned PDMS adhesive pads [179]	Elastic buckling instabilities to switch adhesion states	Temporary double-sided fixation, micro-assembly	High switching efficiency (up to 83%); controlled detachment from one side
Soft hollow pillars (SHPs) with sidewall buckling [181]	Low-pressure control (negative for buckling, positive for bulging)	Microtransfer printing, selective pick-and-place	High adhesion tunability (up to 151×); versatile for varied surface textures and curvatures; low energy cost
Dry adhesive with multiphalange, multifinger design [182]	Buckling ribs for shear load sharing and normal compliance	Robotic manipulators, flexible grippers	High contact area; efficient shear load distribution; adaptable manipulation beyond pick-and-place
Multilayer adhesive with backing, middle, and bottom layers [183]	Preload adjustment for rapid adhesion switching via underside buckling	Transportation, handling applications	High switching ratio (up to 136×); rapid switching; dirt-resistant film-terminated structure
Trigger plant-inspired snap-action metastructure [185]	Tunable spring with snap-through mechanism for adhesion switching	Micro-object handling, pick-and-place systems	Extremely high switching ratio (>10,000); effective in dry/wet and smooth/rough environments
Mushroom-shaped adhesive with magnetized tip [187]	Magnetic actuation for morphology transformation and adhesion switching	Transfer technology, precision pick-and-place	Rapid and reversible adhesion control; noncontact switching for selective pickup and release
Gecko-inspired shape-memory polymer (SMP) adhesive [188]	Shape recovery for reversible adhesion switching	Glass transfer systems, precision assembly	High adhesion (≈332.8 kPa) with easy detachment (3.73 kPa); adaptable to various surfaces
Gecko- and creeper-inspired fibrillar adhesive with PU-GSMP layers [189]	UV-induced photothermal effect for phase change in GSMP micropillars	Robotics, handling rough surfaces	High adhesion strength (278 kPa); high switching ratio (29); fast switching (10 s); adaptable to surfaces with varied roughness
Hierarchical structure with stiffness modulation [190]	Electrothermal film for adjustable stiffness in TPU layer	Soft grippers, wall-climbing robots	High and switchable adhesion on non-flat surfaces; adhesion increased by 10–100× with voltage control
Thermally responsive shape-memory polymer with micropillars [191]	Laser heating for shape recovery to switch adhesion	Noncontact transfer printing, electronics assembly	Strong adhesion for pick-up; laser-driven release for noncontact printing; adaptable to diverse surfaces (e.g., sandpaper, glass)
Superhydrophobic film with shape-memory polyurethane-cellulose nanofiber substrate [192]	Shape-memory effect for switching adhesion states	Controlled droplet manipulation, microfluidics	Captures/releases multiple droplets step-by-step
Shape-memory polymer (SMP) adhesive gripper [194]	Thermoelectric Peltier module for active heating and cooling to switch adhesion	Robotic pick-and-place, manipulation	Strong grip force (>2 atmospheres); minimal release force; works on flat, rough, and wet surfaces
Elastomeric 3D surface structures with conductive nanowire electrodes [197]	Combined shear and electrostatic adhesion, capacitive sensing for touch	Soft grippers, tactile sensors	72% increase in gripping force with voltage; adaptable to various materials; multifunctional for adhesion and force sensing
Gripper with gecko adhesive and thermo-responsive filament [198]	Hydraulic-driven bending actuators, variable stiffness filament	High-load grasping, soft robotics, industrial handling	655% increase in holding force; rapid cooling (31 s); cost-effective and scalable fabrication
Microstructured adhesive with integrated piezoelectric element [199]	Ultrasonic vibration for dust removal and adhesion tuning	Robotics, cleanroom applications	Removes 53–71% of contaminants; recovers 3–11% adhesion post-contamination; maintains performance over 1500 cycles
Combined electrostatic and gecko-inspired adhesive [200]	Tunable adhesion via electrostatic and microstructured adhesive layers	Grasping diverse materials, soft robotics	100–168% increased gripping force across various materials; effective on rough surfaces; adaptable for soft and fragile objects
Hierarchical structure: asymmetric wedges + mushroom fibrils [201]	Loading–dragging–pulling and magnetic field actuation	Flexible devices, releasing ultra-light objects	Dual switchable adhesion modes; overcoming shear-induced adhesion limits; self-cleaning; ultra-light release
Soft gripper with 3D-printed directional adhesives and curved pillars [202]	Adhesion enhancement via additional coating for smoothness and tip deformation	Handling thin, flexible objects (e.g., films, FPCBs)	Cost-effective; environment-friendly; retains 95% adhesion after 10,000 cycles; adhesion recovery with cleaning

## Data Availability

No new data were created or analyzed in this study.
